# Analysis of MicroRNA Expression in Embryonic Developmental Toxicity Induced by MC-RR

**DOI:** 10.1371/journal.pone.0022676

**Published:** 2011-07-29

**Authors:** Yanyan Zhao, Qian Xiong, Ping Xie

**Affiliations:** 1 Donghu Experimental Station of Lake Ecosystems, State Key Laboratory for Freshwater Ecology and Biotechnology of China, Institute of Hydrobiology, The Chinese Academy of Sciences, Wuhan, People's Republic of China; 2 Graduate School of the Chinese Academy of Sciences, Wuhan, People's Republic of China; Kyushu Institute of Technology, Japan

## Abstract

As cynobacterial blooms frequently occur in fresh waters throughout the world, microcystins (MCs) have caused serious damage to both wildlife and human health. MCs are known to have developmental toxicity, however, the possible molecular mechanism is largely unknown. This is the first toxicological study to integrate post-transcriptomic, proteomic and bioinformatics analysis to explore molecular mechanisms for developmental toxicity of MCs in zebrafish. After being microinjected directly into embryos, MC-RR dose-dependently decreased survival rates and increased malformation rates of embryos, causing various embryo abnormalities including loss of vascular integrity and hemorrhage. Expressions of 31 microRNAs (miRNAs) and 78 proteins were significantly affected at 72 hours post-fertilisation (hpf). Expressions of miR-430 and miR-125 families were also significantly changed. The altered expressions of miR-31 and miR-126 were likely responsible for the loss of vascular integrity. MC-RR significantly reduced the expressions of a number of proteins involved in energy metabolism, cell division, protein synthesis, cytoskeleton maintenance, response to stress and DNA replication. Bioinformatics analysis shows that several aberrantly expressed miRNAs and proteins (involved in various molecular pathways) were predicted to be potential MC-responsive miRNA-target pairs, and that their aberrant expressions should be the possible molecular mechanisms for the various developmental defects caused by MC-RR.

## Introduction

Microcystins (MCs), a group of cyclic heptapeptide compounds with specific hepatotoxins produced by cyanobacterial species, have received worldwide concerns in the past decades [1], [2]. So far, more than 80 different structural analogues of MCs have been identified [3], among which MC-LR and -RR are the most common and abundant [4].

Nowadays, accumulating evidence have indicated that MCs have embryonic toxicity in both fish and mammals [5], [6]. The main effects of exposure to MCs in early life stages of fish are interferences with developmental processes and organ functions. The most frequent alteration observed are decrease in survival and growth rate [7], [8] and various embryo abnormalities such as enlarged and opaque yolk sac, small head, curved body and tail, hepatobiliary abnormalities, heart rate perturbations, edema in pericardial sac (PS) and hatching gland (HG) [7], [9], [10], [11].

Embryonic development of animals is strictly regulated at different levels. Accumulating evidence have demonstrated that miRNAs which can regulate gene expression post-transcriptionally by targeting mRNAs, play a fundamental role in early embryonic development [12], [13], [14]. Dicer and Drosha are the miRNA processing enzymes that are required for the maturation of miRNAs [15], [16]. The Dicer knockout mouse did not survive beyond 7.5 days past gastrulation [14]. Dicer-deficient zebrafish arrest during larval development only at around day 10, because maternally contributed Dicer maintains miRNA maturation during the early development of the homozygous mutant [17]. However, if the maternal Dicer contribution is eliminated, defects appear much earlier during gastrulation, brain formation, somitogenesis, and heart development [18].

Mounting evidence have indicated that MCs have developmental toxicity and cause various kinds of abnormalities in early life stages of animals, but the potential molecular mechanism is largely unknown. MiRNAs, which are of vital importance in early embryonic development, are proved to be affected by oxidative and other forms of cellular stress and xenobiotics [19], [20]. Each miRNA species has an effect on the translation of many mRNA species [18] and so a change in its expression level could substantially affect the protein complement of the cell. These facts lead us to consider whether and how miRNA and miRNA-target system contribute to developmental toxicity in animals exposed to environmental pollutants.

Therefore, in this study, we used zebrafish embryo as a model system to investigate the toxic effects of MC-RR on early development, aiming to explore the underlying molecular mechanism at both posttranscriptional and translational levels. Alteration in expression pattern of miRNAs and proteins in embryos treated with MC-RR were detected by miRNA microarray and two-dimensional electrophoresis (2-DE), respectively. We also analyzed the potential contribution of altered miRNAs and their predicted target system to developmental toxicity in embryos of zebrafish after MC-RR exposure. These results would help us better understand the possible molecular mechanisms of embryonic toxicity induced by environmental pollutants and also will guide us to protect human health efficiently.

## Results

### Acute toxicity of MC-RR in zebrafish embryos

To assay the developmental toxicity of MCs, we micro-injected 2 nL MC-RR solution into 2–4 cell stage embryos of zebrafish. Injecting embryos after 1-cell stage allowed us to remove unfertilized eggs easily from our statistics. The LD_50_ value of MC-RR on zebrafish embryos (after incubation for 24 h) was estimated at 36 µM MC-RR dose (injection volume was 2 nL per egg). To analyze the dose-dependent survival of MC-RR, we injected embryos with different doses of MC-RR of 0.2 LD_50_ (7.2 µM), 0.4 LD_50_ (14.4 µM) and 0.8 LD_50_ (28.8 µM).

### Survival and malformation rates

**Figure 1:**
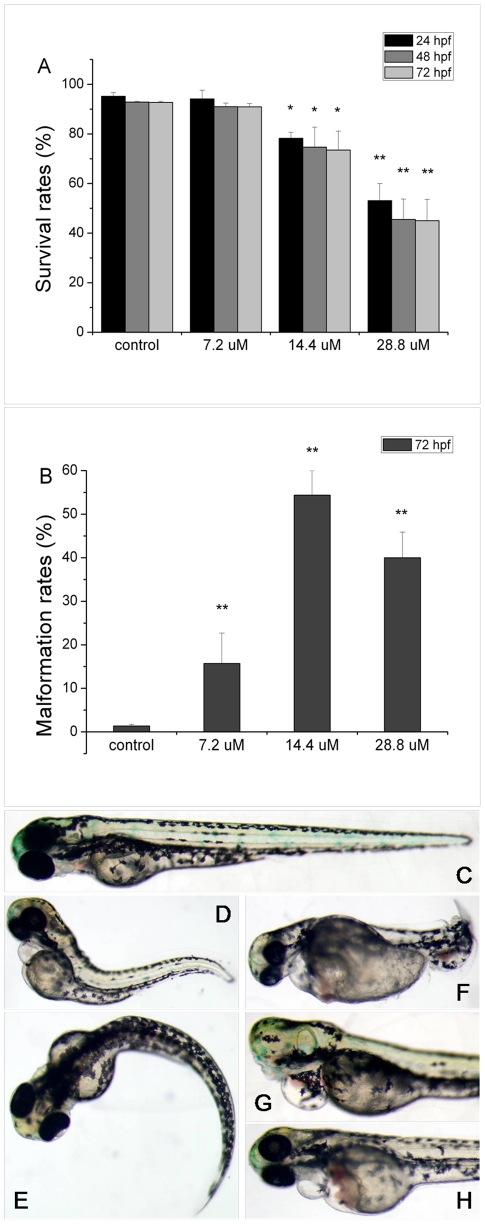
The survival, malformation rates and different developmental defects of zebrafish embryos induced by MC-RR. (A) The survival rates of embryos after MC-RR exposure at 24, 48 and 72 hpf, respectively. (B) The malformation rates of embryos after MC-RR treatment at 72 hpf. Various morphological deformities were detected in MC-RR-treated embryos, including bent tail/body axes (D, E, F), cyclopia (G), as well as edema in pericardial sac (PS) and hatching gland (HG) (H), compared to control embryos (C). Values are expressed as mean ± SD (*indicates significant change compared to control at *p*<0.05, **indicates significant change compared to control at *p*<0.01).

At 24, 48 and 72 hpf, we examined the survival rates of zebrafish embryos treated with different concentrations of MC-RR (7.2 µM, 14.4 µM and 28.8 µM). As shown in Figure 1A, the survival rates of embryos decreased with the elevation of MC-RR concentration from 7.2 µM to 28.8 µM, but there were no obvious changes at 72 hpf compared with 48 hpf at any dose of MC-RR. Marked toxic effects (*p*<0.01) on malformation of larvae were observed at 72 hpf after exposure of MC-RR, with malformation rates of 15.7%, 54.4% and 39.9% in 7.2 µM, 14.4 µM and 28.8 µM, respectively (Figure 1B). The embryos treated with MC-RR exhibited a consistent and highly reproducible pattern of morphological abnormalities, and defects were most frequently observed in tails. Tails were bent (Figure 1D), curving (Figure 1E) or twisting (Figure 1F). In some cases, cyclopia occurred as shown in Figure 1G. Figure 1H shows a fry with edema in pericardial sac (PS) and hatching gland (HG). Table 1 shows the percentages of particular defects in the different MC treatments.

**Table 1:** Percentages of particular defects in the different MC treatments.

	Bent tails (%)	Curving tails (%)	Twisting tails (%)	Cyclopia (%)	Edema in PS (%)	Edema in HG (%)
Control	0	0.7±1.1	0	0	0	0
7.2 µM	4.9±1.4	5.6±2.3	2.8±1.3	0	13.0±2.0	13.0±1.0
14.4 µM	18.0±2.0	19.0±3.0	14.0±3.0	5.0±2.0	20.0±2.0	23.0±3.0
28.8 µM	9.0±3.0	11.0±3.0	25.0±5.0	3.0±2.0	22.0±7.0	25.0±2.0

Note: PS means pericardial sac and HG means hatching gland.

According to the dose and time-dependent survival and malformation status of embryos after MC-RR exposure, embryos treated with 14.4 µM MC-RR (approximately 0.0288 ng MC-RR per egg) at 72 hpf were selected for the miRNA microarray and 2-DE assay. The concentration of MC-RR was selected on the basis of the highest malformation rate and the most diverse types of embryos abnormalities with relatively low mortality after MC-RR treatment.

### MC-RR exposure decreased the number of complete intersegmental vessels (ISVs)

**Figure 2:**
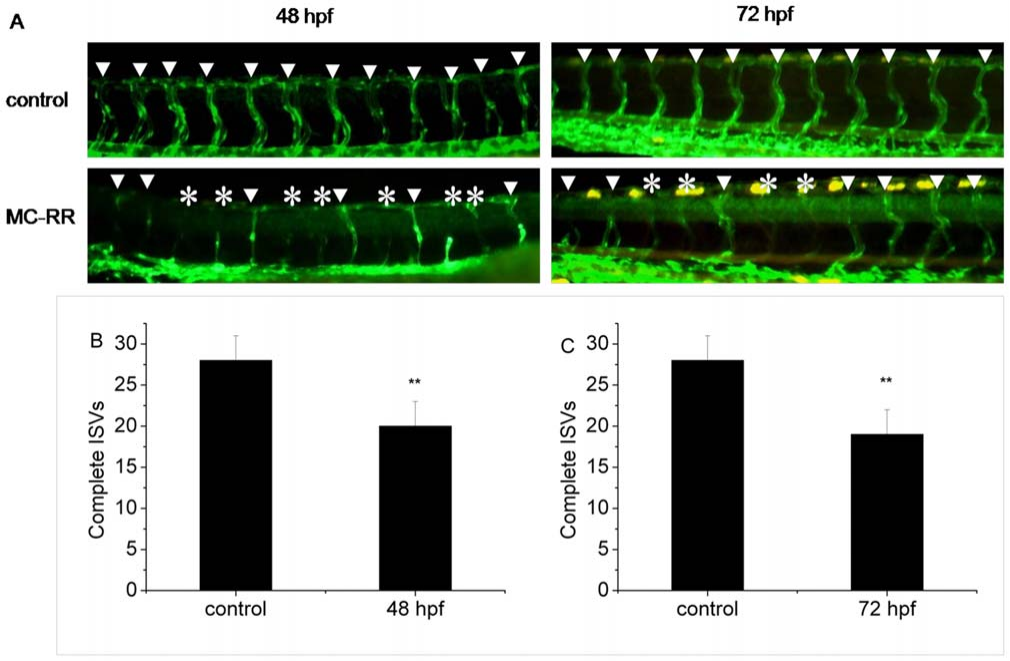
MC-RR exposure decreased the number of complete ISVs. Treated with MC-RR resulted in a significant reduction in the number of complete ISVs at 48 hpf (A and B) and 72 hpf (A and C), respectively, compared with controls. Arrowheads indicate complete ISVs. Asterisks indicate incomplete ISVs. (**indicates significant change compared to control at *p*<0.01).

To determine the toxicity of MC-RR to the development of zebrafish blood vessel, we used Tg-(flk:GFP) zebrafish embryos, in which the angiogenesis is clearly visible. Sprouting was quantified at 48 hpf and 72 hpf following the injection of 14.4 µM of MC-RR or 0.8% saline solution. At 48 hpf, when most ISVs in the control embryos had fully extended dorsally to form the dorsal longitudinal anastomotic vessel (DLAV), MC-RR exposure resulted in a significant reduction in the number of complete ISVs (Figure 2A and Figure 2B, *p*<0.01, n = 30). This deficit in ISV formation was maintained at 72 hpf (Figure 2A and Figure 2C, *p*<0.01, n = 30).

### miRNA expression profile in MC-RR treated zebrafish embryos

**Figure 3:**
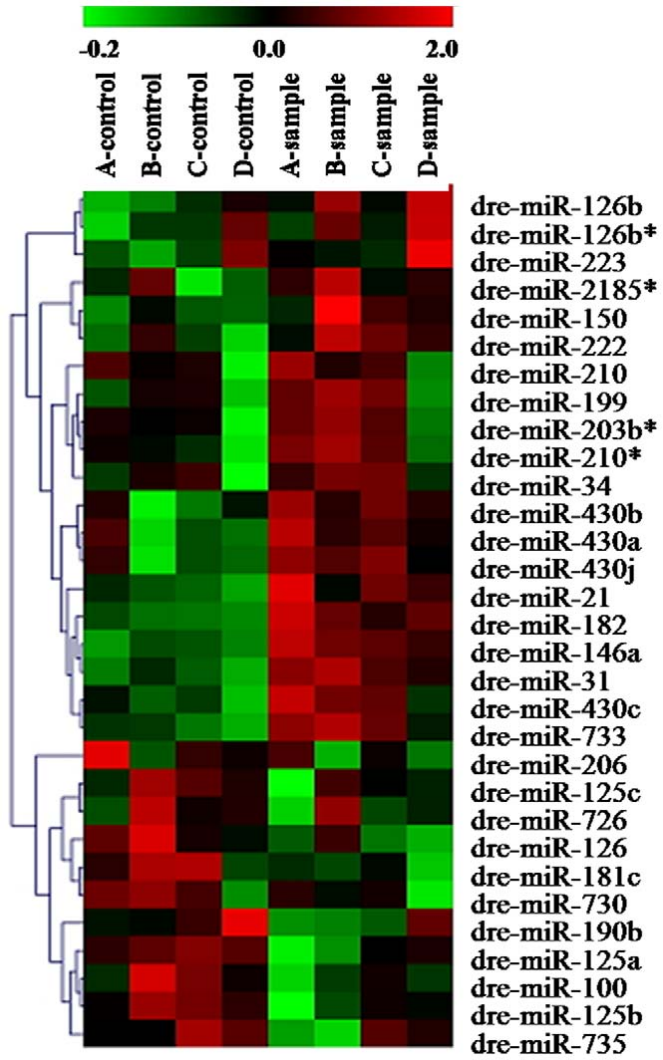
Hierarchical clustering of the differentially expressed miRNAs in control and MC-RR-treated zebrafish embryos. Each row represents one miRNA with significantly differential expressions between control and treatment (*p*<0.05). Each column represents a biological replicates; in each panel, the left four columns are for controls and the right four for MC-RR treatments. Colors represent expression levels of each individual miRNA: red, up-regulation; green, down-regulation.

To investigate whether and what miRNAs expression might be regulated by MC-RR, we analyzed the miRNA expression profile of zebrafish embryos treated with MC-RR by using an array-based miRNA profiling. A comparison of miRNA expression levels between control and MC-RR-treated zebrafish embryos revealed that MC-RR-exposure significantly altered miRNA expression profiles (Figure 3).

The expression levels of 31 miRNAs were significantly altered in zebrafish embryos after MC-RR exposure (Table 2). Of the 31 miRNAs, 20 were significantly up-regulated and 11 were evidently down-regulated (*p*<0.05). However, the extent of changes varied among miRNAs. Dre-miR-146a exhibited the largest effect size (6.73-fold upregulation) in MC-RR exposed zebrafish embryos relative to controls, whereas the expression of dre-miR-190b showed the largest downregulation (4.61-fold).

**Table 2:** 31 differentially expressed miRNAs induced by MC-RR and their predicted potential target proteins (*p*<0.05).

miRNA	miRNA fold change	Target symbol	Protein fold change
dre-miR-146a	6.73	atpb	2.77
		sri	0.39
dre-miR-430c	3.69	atpb	2.77
		cct3	2.23
		ppa1	0.48
dre-miR-21	3.04	-	-
dre-miR-223	2.57	-	-
dre-miR-733	2.55	-	-
dre-miR-31	2.14	cct3	2.23
		pkm2b	0.50
dre-miR-430a	2.08	atp5a1	0.23
		cyt1	1.97
dre-miR-430j	1.92	atp5a1	0.23
		pkm2b	0.50
dre-miR-430b	1.76	pkm2b	0.50
		cyt1	1.97
dre-miR-2185*	1.67	-	-
dre-miR-150	1.58	tubb5	3.47
		ppp2r1a	0.51
dre-miR-210*	1.48	-	-
dre-miR-199	1.44	-	-
dre-miR-182	1.42	txnl1	0.49
dre-miR-203b*	1.42	-	-
dre-miR-210	1.41	-	-
dre-miR-126b	1.29	-	-
dre-miR-222	1.28	-	-
dre-miR-34	1.22	-	-
dre-miR-126b*	1.19	-	-
dre-miR-730	0.76	rpsa	0.48
dre-miR-726	0.73	-	-
dre-miR-206	0.67	bhmt	0.51
dre-miR-126	0.65	-	-
dre-miR-100	0.65	-	-
dre-miR-181c	0.64	rpsa	0.48
		ppp2r1a	0.51
dre-miR-735	0.62	-	-
dre-miR-125c	0.58	hspa8	0.44
dre-miR-125b	0.50	hspa8	0.44
dre-miR-125a	0.47	ppa1	0.48
dre-miR-190b	0.21	acta1	1.98

### Confirmatory studies on differentially expressed miRNAs by Quantitative Real-Time PCR (qPCR)

**Figure 4:**
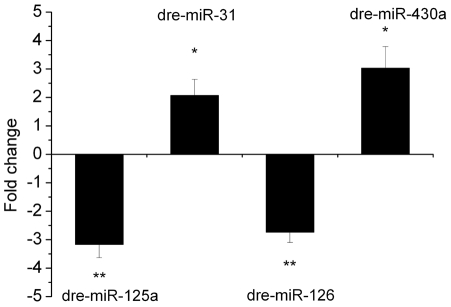
Validation of differentially expressed miRNAs in MC-RR treated embryos comparing with controls. Values represent the mean±SD of three independent samples, each run in triplicate (*indicates significant change at *p*<0.05, **indicates significant change at *p*<0.01).

To validate the microarray data, we assayed expression levels of four miRNAs (dre-miR-125a, dre-miR-126, dre-miR-31, and dre-miR-430a) by qPCR and compared the results from the microarray and qPCR. Of the miRNAs selected for comparison, two miRNAs (dre-miR-31 and dre-miR-430a) were up-regulated whereas two miRNAs (dre-miR-125a and dre-miR-126) were down-regulated based on the results of microarray analysis. The expression data obtained by qPCR analysis are comparable with the microarray analysis data (Figure 4).

### Overexpression of miR-126 in MC-RR treated zebrafish embryos

**Figure 5:**
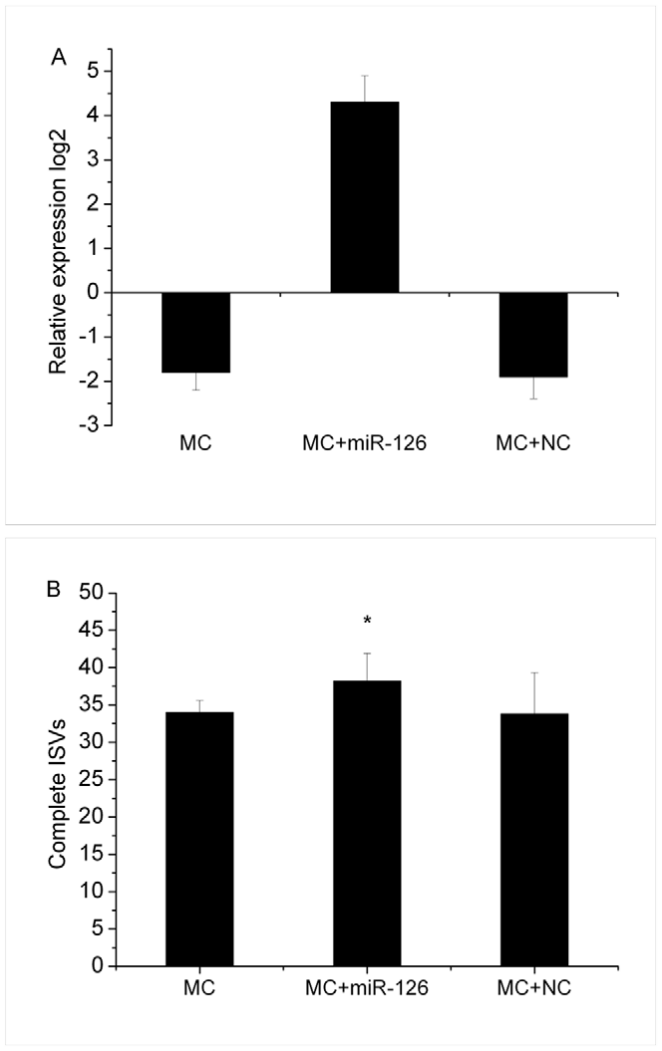
MiR-126 overexpression in MC-RR treated zebrafish embryos. (A) The relative expression level of miR-126 in three groups (MC-RR group, MC-RR+miR-126-DP group and MC-RR+NC group). (B) miR-126-DP co-injected with MC-RR into embryos could slightly rescue the ISV. Values represent the mean±SD of three independent samples, each run in triplicate. (*indicates significant change compared to control at *p*<0.05).

MiR-126 which has been proved to contribute to the ISVs formation was chosen for further study. The expression of miR-126 is detected in the three groups (MC-RR group, MC-RR+miR-126-DP group and MC-RR+NC group) at 48 hpf. Coinjection of miR-126 duplex (5 µM) with MC-RR resulted in an almost 19-fold increase of miR-126 levels compared with controls, while the expression of miR-126 in MC-RR+NC group showed no significant alteration in miR-126 expression level compared to MC-RR group (Figure 5A). MiR-126-DP co-injected with MC-RR into embryos could slightly rescue the ISV formation. Coinjection resulted in just a little increase (∼8%) in the number of complete ISVs compared with MC-RR groups (Figure 5B).

### 2-DE and mass spectrometry identifies proteins affected by MC-RR injection

**Figure 6:**
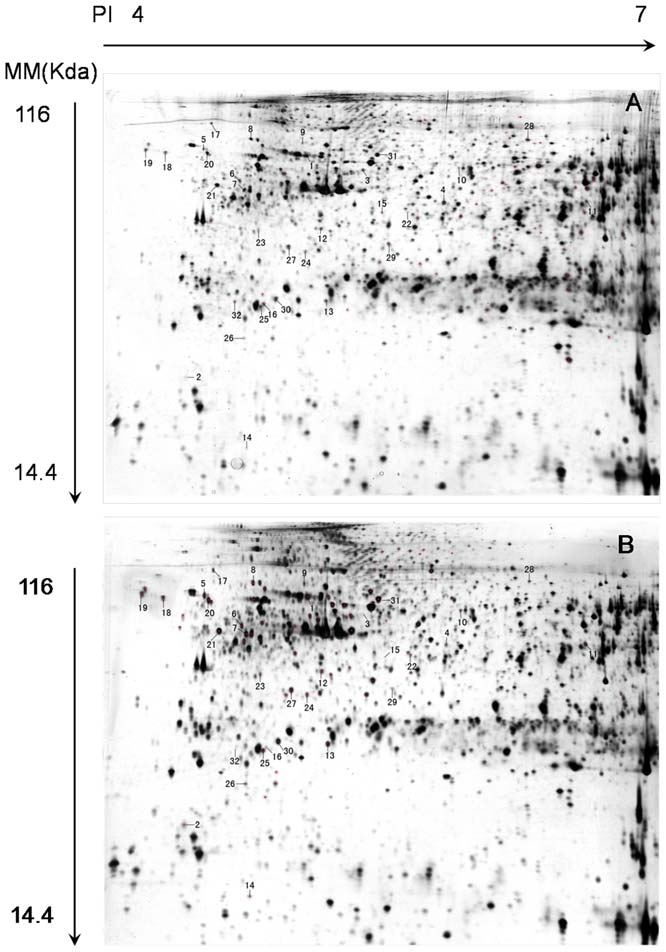
Representative 2-DE gels of the protein profiles obtained from the MC-RR-treated zebrafish larvae 72 hpf. (A) 2D gel image with proteins expressed in the control condition; (B) 2D gel image with proteins expressed in the MC-RR-treated condition. The proteins of the samples were separated on a pH 4–7 liner IPG strip, followed by 12.5% SDS-polyacrylamide gel and silver staining. The cross and number indicate spots with significant changes in intensity (*p*<0.01, Student's t-test in three independent gels). Each experiment was conducted independently at least three times, and an image taken from one representative experiment is shown.

Most miRNAs could bind to their target mRNAs imperfectly to repress protein translation. In this study, protein level was investigated by 2-DE and MALDI-TOF-MS /MS analysis. A twofold change cutoff was used as the criterion for differential expression of proteins. Compared with the gels from the controls, 78 protein spots were found to have been significantly altered by the effects of MC-RR. A total of 38 protein spots were selected for protein identification based on spot intensity and spot integrity (Figure 6). After careful manual extraction, the protein spots were subjected to MALDI-TOF-mass specrometry (MS)/MS analysis. After a PMF search in the NCBI nr database, 32 spots were successfully identified. MC-RR exposure resulted in up-regulation of 7 proteins and down-regulation of 25 proteins. The protein codes, accession numbers, descriptions, and fold changes are listed in Table S1.

## Discussion

As toxic cyanobacterial blooms frequently occur in lakes, rivers or ponds throughout the world, the potential risk of MCs exposure to pregnant women and their offspring should not be neglected. In metazoans, early embryonic patterning and organ morphogenesis involve complex cellular, and developmental processes depend on precise spatiotemporal regulation of mRNA and protein levels of key regulatory factors [21]. Animal development is an extremely robust process resulting in stereotyped outcomes. So when the extremely robust process was disturbed, developmental defects of animals may occur. Regulation by miRNAs was considered to play essential roles in embryonic development [22], [23], [24]. Giraldez et al. [18] demonstrated that absence of mature miRNAs leads to morphological defects and the subsequent death of larvae. So it becomes intriguing whether and how miRNAs contribute to abnormal development in animals exposed to environmental pollutants.

In the present studies, after microinjected MC-RR directly into embryos, we observed that MC-RR dose-dependently decreased the survival rates and increased malformation rates of embryos, and that MC-RR caused various embryo abnormalities including loss of vascular integrity and hemorrhage. MC-RR has specific toxicity to liver of adults, however, its influence on liver development in embryos of zebrafish was not obviously detected in current study. These suggest that the adverse effects of MC-RR on embryos of zebrafish were multiple and complicated and differed with developmental stages. These developmental defects indicate that MC-RR affected upstream factors mastering the embryonic development and subsequent organogenesis [11].

This study is the first to find MC-responsive miRNAs in animals. We observed significantly changed expressions of 31 miRNAs in embryo of zebrafish exposed to MC-RR at 72 hpf. A number of studies have demonstrated the important roles of particular miRNAs in specific processes during development in zebrafish though most miRNAs functions are still unknown. In the present studies, expression changes in miR-430 and miR-125 families were quite significant. Giraldez et al. [18] observed that injection of miR-430 rescues the early morphogenesis defects in dicer mutants. So the high expression of miR-430 family here may be the organism's strategy to rescue the impairment or to balance the disorders of miRNAs and their target system caused by MC-RR. It has been demonstrated that miR-125b is an important negative regulator of p53 and p53-induced apoptosis during development as well as in stress response [25]. The aberrant expression of these typical miRNAs in the present study indicates that MC-RR has significant influence on these important regulation factors and certainly on most cellular process during development of embryos.

**Figure 7:**
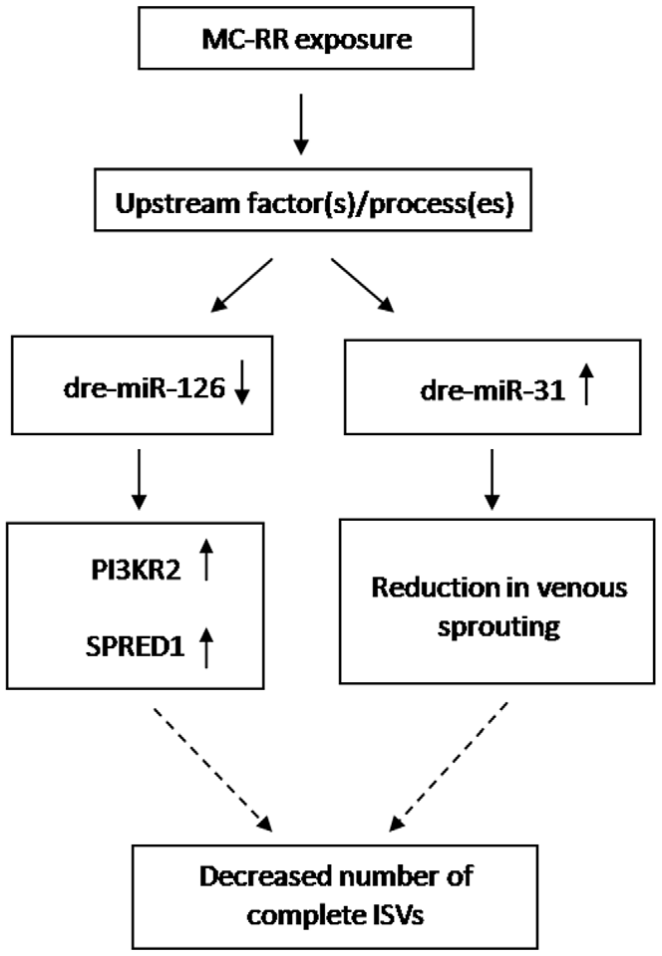
A proposed model showing the mechanisms of MCs-induced vascular defects. MC-RR exposure caused aberrant expression of dre-miR-126 and dre-miR-31. Downregulation of dre-miR-126 can resulted in the upregulation of PI3KR2 and SPRED1, two negative regulators of the VEGF signaling pathway, upregulation of dre-miR-31 can lead to reduced venous sprouting. Dre-miR-126 and dre-miR-31 pathways may together cause significantly decreased number of complete ISVs in zebrafish embryos. In this model, continuous lines show steps confirmed in this study or elsewhere; the dashed lines represent processes that have not been proved by direct evidence.

We also for the first time detected the decreased number of complete ISVs in embryos after MC-RR exposure. MiR-31 and miR-126, two miRNAs that have been proved to contribute to vascular development were significantly altered in the present study. Pedrioli et al. [26] observed that over-expression of miR-31 reduce venous sprouting of zebrafish embryo. MiR-126 regulates vascular endothelial growth factor (VEGF)-dependent PI3 kinase and MAP kinase signaling by directly targeting PI3KR2 and SPRED1, two negative regulators of the VEGF signaling pathway, respectively, knockdown of miR-126 in zebrafish results in loss of vascular integrity and hemorrhage during embryonic development [27]. Thus, in the present study, the upregulation of miR-31 and downregulation of miR-126 may be related to the vascular defects of embryos caused by MC-RR exposure (Figure 7). Furthermore, to verify this hypothesis, we performed a rescue experiment for testing whether the deficit in ISV formation is related to the altered expression of miR-126 induced by MC-RR. We co-injected miR-126 duplex with MC-RR into the embryos and found that miR-126 overexpression could only slightly rescue the ISV phenotype. As we known, miRNAs that have been identified in zebrafish is very limited and the function of most miRNAs still remains unknown. It is possible that except miR-126 and miR-31 there are other known or unknown miRNAs contributing to the formation of ISVs. All miRNAs are required but not sufficient individually to precisely regulate the ISVs formation. In addition, other factors besides miRNAs might have contributed together to the defects of ISV formation post MC-RR exposure. So this is an intriguing question and further researches will be done in our future work.

Although mRNAs are the direct targets of miRNA, it is of great importance to further consider the alteration of the final product of gene regulation - protein. MC-RR significantly reduced the expression of a number of proteins involved in energy metabolism, cell division, protein synthesis, cytoskeleton maintenance, response to stress and DNA replication. Several proteins were predicted to be regulated by the differentially expressed miRNAs after MC-RR treatment. Among these putative MC-responsive miRNA-target pairs, 9 pairs of miRNAs and proteins were coherently expressed (e.g. miRNA expression was active and target transcription was repressed) while other 15 pairs were incoherently expressed (e.g. both miRNA expression and target transcription were repressed) (Table 2). This could be due to the different architectures of miRNA-target network and the different regulation of transcription factors [28]. The relationship between protein expression and miRNA regulation may be complicated. One miRNA could regulate the expressions of a distinct set of proteins, and meanwhile a protein may be regulated by several miRNAs. So the expression level of a protein can be affected by the combined effects of several miRNAs, some of which may be up-regulated (that will result in the reduction of protein expression) and the others may be down-regulated (which may increase the expression of protein) after toxin treatment. So the combined effects of these miRNAs to the expression of one protein are quite uncertain. In addition, the expression of proteins in embryos could also be affected by other factors after toxin treatment in the present study. So, although a number of miRNAs and proteins affected by MC-RR were computationally predicted to be potential target pairs, the expressions of proteins and related miRNAs could still be coherent or incoherent. These proteins and the related miRNAs affected by MC-RR are involved in regulation of multiple signaling pathways mainly including metabolism and cell signaling, such as glycolysis, oxidative phosphorylation, glycine, serine and threonine metabolism and Wnt signaling (Table 3). Wnt signaling is demonstrated to be indispensable for orchestrating the complex cell behaviors that occur throughout development [29]. At the cellular level, all developmental processes are ultimately controlled by the cooperative actions of different signal transduction pathways [30]. Therefore, altering the expression of miRNAs involved in the regulation of various molecular pathways might be the very first and crucial event in embryos in response to MC exposure. The putative miRNA and target proteins pairs may offer a special MC-responsive biomarker in embryos.

**Table 3:** List of pathways involved in MC-RR induced toxicity in embryos.

Pathways	p-Value	Proteins
Cell communication	2.16E-07	Krt4; Krt5; Cyt1
Carbon fixation	1.50E-05	Pkm2b; Pgk1
Glycolysis	6.90E-05	Pkm2b; Pgk1
Ribosome	8.63E-05	Rpsa; Rplp0
Cytoskeletal regulation by Rho GTPase	1.72E-04	Tubb2c; Tubb5
Oxidative phosphorylation	3.89E-04	Atp5a1; Atpb
Wnt signaling pathway	6.54E-04	Ppp2r1a; Gnb1l
Glycine, serine and threonine metabolism	0.005593	Bhmt
Methionine metabolism	0.005836	Bhmt
Pyruvate metabolism	0.007532	Pkm2b
TGF-beta signaling pathway	0.019571	Ppp2r1a
Purine metabolism	0.022917	Pkm2b
MAPK signaling pathway	0.040434	Hspa8

The toxicity pathways were involved by differentially expressed proteins induced by MC-RR with *p*-values<0.05.

Based on the above results and discussion, we suggest that miRNA and their miRNA-target circuitry disturbance may contribute to MC-RR caused developmental toxicity. Then how this happened? There are two possible mechanisms. First, miRNAs may work similarly as developmental switches so that people could necessarily observe a striking phenotype in the absence of miRNAs [28]. Such as miRNA-126 and miRNA-31 that are demonstrated to be crucial for the vascular development in embryos of zebrafish. Second, miRNAs may have other common functions, serving a different purpose from primary gene regulation. Most differentially expressed miRNAs and potential target proteins caused by MC-RR might have these functions. These two types of mechanisms may work together to regulate the whole developmental process of embryos. Therefore, when the milieu of miRNA and the target system is disturbed, the genetic network may be challenged. That is possibly why we observed the developmental toxicity in such a wide manner in embryos of zebrafish after MC-RR exposure. MCs may influence animal development through diverse regulatory pathways. Bartel and Chen [31] pointed out that post-transcriptional control could be more responsive than transcriptional control. It is suggested that miRNA-target circuitry has evolved under stabilizing selection to provide robustness to genetic networks for reducing phenotypic variability in the population [28], [32]. Thus, disturbing miRNA-based gene-regulatory system would be more powerful for MCs to influence developmental processes of animals.

### Conclusion

We for the first time integrated post-transcriptomic, proteomic and bioinformatics analysis to explore molecular mechanisms for the toxicity of MCs on embryo of zebrafish. MC-RR dose-dependently decreased survival rates and increased malformation rates of embryos. Several aberrantly expressed miRNAs and the proteins were predicted to be potential MC-responsive miRNA target pairs by bioinformatics analysis, which are directly or indirectly involved in various molecular processes/pathways and are crucial for embryonic development. These provide a molecular mechanism for the embryonic developmental toxicity of MCs and may be helpful to predict risk of MCs to human health in the future.

## Materials and Methods

### Zebrafish maintenance and embryo collection

Mature wild-type AB strain and Tg-(flk:GFP) zebrafish (about 8 month old) were maintained at 28±0.5°C in a 14 h: 10 h light: dark cycle in a continuous flow-through system in charcoal-filtered tap water. The fish were fed twice daily with *Artemia nauplii.* Embryos were collected at 15–20 min intervals after spawning, washed and incubated in Ringer's solution (116 mM NaCl, 2.9 mM KCl, 1.8 mM CaCl_2_ and 5 mM HEPES) at 28.5°C until use. All chemicals were from Sigma (St Louis, MO) unless otherwise stated.

### MC-RR exposure

MC-RR (purity >95%) standards was purchased from Sigma Chemical (St. Louis, MO, U.S.A.), dissolved in 0.8% saline solution at different concentrations. To determine Median lethal dose (LD_50_) and to select relevant MC-RR exposure dose for the following experiments, zebrafish embryos were exposed to MC-RR by microinjection at a range of doses (12–48 µM). Three replicate treatments (3×30 embryos) were exposed to each dose. Observations for morphologic effects were made; any dead larvae were counted and removed. The average proportion of larvae responding for a given end point was calculated for each dose. LD_50_ was calculated from a linear regression of log-probit transformations of the dose-response data [33]. All of the experimental researches on zebrafish were performed with the approval of the animal ethics committee in the Institute of Hydrobiology, Chinese Academy of Sciences (Study ID#O91105-1-201).

### Microinjection procedures

Glass capillaries of 1.14 mm O.D. and 0.5 mm I.D. (World Precision Instrument Inc., Sarasota, FL) were pulled using a horizontal puller (P-97, Sutter Instrument, Navato, CA). Embryos at desired stages as judged according to Kimmel et al [34] were immobilized at an injection trough on a 100 mm 2% agar plate. We microinjected 2 nL volume of MC-RR into the yolk of 2–4 cell stage embryos by using a picoliter injector (Harvard instruments). After injection, embryos were recovered from injection troughs and cultured in Ringer's solution at 28.5°C until use.

### Effect of MC-RR on survival and malformation

To analyze the dose-dependent survival and malformation status of MC-RR, we injected embryos with different doses of MC-RR of 0.2 LD_50_, 0.4 LD_50_ and 0.8 LD_50._ The time point at which specific embryonic structures were formed were compared with those described in an established atlas for normal zebrafish development [34]. The embryos were examined under a Leica mz125 microscope at 24, 48 and 72 hpf to screen for morphological abnormalities, and the survival status was recorded on the total of 30 embryos initially in each treatment.

### Protein Extraction

Protein extraction was performed according to Shi et al [35] with slight modification. At 72 hpf, approximately 200 zebrafish larvae were homogenized by a power homogenizer in 500 µl lysis buffer containing 7 M urea, 2 M thiourea, 4% 3-[(3-chola-midopropyl) dimethylammonio]-1-propanesulfonate (CHAPS), 1% wt/vol dithiothreitol (DTT), 40 mM Tris base, 1% protease inhibitor cocktail, 0.5 µl benzonase (25 U/µl, >99% purity; Novagen, Madison, WI), and 20 µl/ml Bio-Lytes 3/10. Then, the samples were disrupted by intermittent sonic oscillation for 5 min and incubated on a shaker for 30 min at 4°C. Insoluble particles were removed by centrifugation at 12,000×g for 1 hour at 4°C, then the supernatants were collected. Four volumes of 100% ice-cold acetone were added into 1 vol of supernatant. The samples were precipitated at −20°C for 1 h and then centrifuged at 12,000×g for 15 min at 4°C. The supernatants were discarded and the protein pellets were dissolved in a protein solution buffer (7 M urea, 2 M thiourea, 4% CHAPS, 1% wt/vol DTT, and 0.5% Immobilized pH Gradient (IPG) buffer). Protein concentrations were determined using the Bradford assay [36].

### 2-DE analysis

350 µg of each protein sample was mixed with a rehydration buffer (7 M urea, 2 M thiourea, 4% CHAPS, 1% wt/vol DTT, 0.5% IPG buffer, and a trace of bromophenol blue) and then loaded onto IPG strips (pH 4–7, 18 cm, Bio-Rad). Isoelectric focusing (IEF) was performed in IPG strips (pH 4–7, 18 cm, Bio-Rad) on a PROTEAN IEF cell system (Bio-Rad). After the IEF program, the strips were equilibrated in an IPG equilibration buffer (6 M urea, 2% SDS, 30% glycerol, 0.375 M Tris, pH 8.8, 20 mg/ml DTT, and a trace of bromophenol blue) and then alkylated (25 mg/ml iodoacetamide instead of DTT in an equilibration buffer) for 15 min each. Subsequently an 11.25 % SDS-PAGE second dimension was performed with a PROTEAN II MULTIP CELL (Bio-Rad). Electrophoresis was carried out at 20 milliamperes per gel for 40 min, followed by separation at 30 milliamperes per gel until the dye front had nearly reached the bottom. The protein spots were visualized via silver staining. Two-dimensional gels were performed in triplicate and from three independent protein extractions for each group.

### Image Acquisition and Analysis

The gel images were captured on a Calibrated Densitometer GS-800 (Bio-Rad). PDQuest gel image analysis (Bio-Rad) software was used to match and analyze the images. The protein spots were detected automatically and then edited manually to remove streaks, speckles, and artifacts. The quantification of the proteins was expressed as the volume of spots, which was determined in comparison with the total volume of all the spots within the gel. Only protein spots showing a significance (*p*<0.05) and at least a 2.0-fold difference in abundance (ratio of mean normalized spot volume of treated *versus* control groups) were considered as up- or down- regulated.

### Mass spectrometric analysis

The protein spots of interest were selected and washed twice using 200 mM ammonium bicarbonate in 50% acetonitrile/water for 45 min at 37°C, then dehydrated using acetonitrile and spun dry. The dried gel bands were rehydrated in a minimal volume of 25 mM ammonium bicarbonate buffer that contained 10 ng/µL modified trypsin (Promega, Madison, WI) and incubated overnight at 37°C. Matrix-assisted laser desorption/ionization-time of flight (MALDI)-TOF experiments were performed on an Ultraflex TOF-TOF instrument (Bruker Daltonic, Bremen, Germany). The instrument was set in re flector mode. Protein identification was carried out using a combination of PMFs and peptide fragmentation patterns as inputs to search the National Center for Biotechnology Information (NCBI) nonredundant (nr) database using the Mascot search engine (www.matrixscience.com).

### MiRNA microarray assay

Microarray assay was performed using a service provider (LC Sciences). The assay started from 2 to 5 µg total RNA sample, which was size fractionated using a YM-100 Microcon centrifugal filter (from Millipore) and the small RNAs (<300 nt) isolated were 3′-extended with a poly(A) tail using poly(A) polymerase. An oligonucleotide tag was then ligated to the poly(A) tail for later fluorescent dye staining; Hybridization was performed overnight on a µParaflo microfluidic chip using a micro-circulation pump (Atactic Technologies). On the microfluidic chip, each detection probe consisted of a chemically modified nucleotide coding segment complementary to target miRNA (containing 248 unique zebrafish mature miRNAs from miRBase, http://microrna.sanger.ac.uk/sequences/) and other RNA (50 controls and 99 customer defined sequences) and a spacer segment of polyethylene glycol to extend the coding segment away from the substrate. The detection probes were made by in situ synthesis using PGR (photogenerated reagent) chemistry. The hybridization melting temperatures were balanced by chemical modifications of the detection probes. Hybridization used 100 µL 6xSSPE buffer (0.90 M NaCl, 60 mM Na_2_HPO_4_, 6 mM EDTA, pH 6.8) containing 25% formamide at 34°C. After hybridization detection used fluorescence labeling using tag-specific Cy5 dyes. Hybridization images were collected using a laser scanner (GenePix 4000B, Molecular Device) and digitized using Array-Pro image analysis software (Media Cybernetics). Data were analyzed by first subtracting the background and then normalizing the signals using a LOWESS filter (Locally-weighted Regression). We have deposited the raw data at GEO under accession number GSE29329, we can confirm all details are MIAME compliant.

### MiRNA expression analysis by qPCR

MiRNA expression was quantified using the NCode™ SYBR® Green miRNA RT-PCR kit (Invitrogen) according to the manufacturers' instructions. One microgram of total RNA was used for cDNA synthesis. Reverse transcriptions were carried out in triplicate and analyzed using a Chromo4 Real-Time Detection System (MJ Research, Cambridge, MA). The relative quantification values for each miRNA were calculated by the 2^−ΔΔCt^ method [37] using 5S rRNA as an internal reference. The efficiencies of the two primer sets are approximately equal and that they are close to 1.

### MiR-126 overexpression in MC-RR treated zebrafish embryos

MiR-126 was previously proved to contribute to vascular development. Therefore, we performed a rescue experiment for testing whether the deficit in ISV formation is related to the altered expression of miR-126 induced by MC-RR. Synthetic miRNA duplex of miR-126 (miR-126-DP) and negative control miRNA duplex (NC) were purchased from Dharmacon (Dharmacon Inc., Lafayette, CO, USA). MiR-126-DP and NC sequences are UCGUACCGUGAGUAAUAAUGC and UUGUACUACACAAAAGUACUG. For miR-126 overexpression, three different groups were set for microinjection: 1. MC-RR; 2. MC-RR+miR-126-DP; 3. MC+NC group. MiR-126-DP and NC-DP were co-injected with MC-RR into one-cell-stage of Tg-(flk:GFP) zebrafish embryos, respectively. MC-RR was injected at 14.4 µM; MiRNA duplexes were injected at 4–10 µM. For each group, ∼100 eggs were injected, and the embryos with complete ISVs were counted under the fluorescence micrcscope. All experiments were performed in triplicate and the number of ISVs was counted on the total of 50 embryos initially in each group. The quantitative RT-PCR was performed to determine the expression levels of miR-126 in each group.

### Bioinformatics and Statistical Analysis

The classification and functions of the proteins identified were obtained by searching Gene Ontology (www.geneontology.org). Predicted mRNA targets were obtained by combining a variety of currently available prediction algorithms miRanda (http://cbio.mskcc.org/cgi-bin/mirnaviewer/mirnaviewer4.pl) and miRBase (http://www.mirbase.org/). Toxicity pathways were identified by using KEGG PATHWAY (http://www.genome.jp/kegg/pathway.html), PANTHER (http://www.pantherdb.org/genes/) and molecule annotation system (MAS 3.0) (http://bioinfo.capitalbio.com/mas3/).

A two-tailed Student's t-test was used to determine the significant differences between the control and exposure groups. Statistical analysis was performed using SPSS 13.0 software (SPSS, Chicago, IL), and *p*<0.05 was considered to be a statistically significant difference.

## Supporting Information

Table S1 Altered expression of proteins in embryos of zebrafish after MC-RR treatment.
(DOC)
