# Supplementary material for: Analysis of MicroRNA Expression in Embryonic Developmental Toxicity Induced by MC-RR
Source: PLoS One. 2011 Jul 29;6(7):e22676. doi: 10.1371/journal.pone.0022676 (PMC3146480; doi:10.1371/journal.pone.0022676)
Supplement: Table S1 — Altered expression of proteins in embryos of zebrafish after MC-RR treatment. (DOC) [file pone.0022676.s001.doc]

**Table S1. Altered expression of proteins in embryos of zebrafish after MC-RR treatment.**

| **No. on gel** | **Accession no.** | **Identification** | **Fold change** | **Mw (kDa):** | **pI** | **Sequence coverage %** | **Score** | **Decription** |
| --- | --- | --- | --- | --- | --- | --- | --- | --- |
| 1 | gi|41054233 | Tubulin, alpha4 like (Tuba4l) | 0.50 | 50.92 | 5 | 17% | 123 | microtubule-based process, microtubule-based movement |
| 2 | gi|295314924 | Tubulin beta  (Tubb2c) | 0.18 | 6.933 | 9.9 | 55% | 195 | microtubule-based process, microtubule-based movement |
| 3 | gi|38488747 | Tubulin, beta 5  (Tubb5) | 3.47 | 50.1 | 4.8 | 11% | 93 | microtubule-based process, microtubule-based movement |
| 4 | gi|18858249 | Actin, alpha 1  (Acta1) | 1.98 | 42.29 | 5.2 | 26% | 389 | muscle thin filament assembly; skeletal muscle fiber development |
| 5 | gi|94733727 | Tumor rejection antigen (Gp96) 1  (Hsp90b1) | 0.19 | 91.4 | 4.8 | 16% | 404 | protein folding, response to stress |
| 6 | gi|226823315 | hsp90ab1 Heat shock protein HSP 90-beta  (Hsp90ab1) | 0.33 | 83.7 | 4.9 | 17% | 452 | protein folding, response to stress |
| 7 | gi|160333682 | Heat shock cognate 71 kDa protein  (Hspa8) | 0.44 | 71.16 | 5.2 | 17% | 386 | response to stress |
| 8 | gi|148725496 | Protein phosphatase 2 (Formerly 2A), regulatory subunit A, beta isoform  (Ppp2r1a) | 0.51 | 66.18 | 4.9 | 29% | 360 | binding |
| 9 | gi|41393119 | Transitional endoplasmic reticulum ATPase  (Vcp) | 0.38 | 90 | 5.1 | 19% | 97 | nucleotide binding, ATP binding, hydrolase activity, nucleoside-triphosphatase activity |
| 10 | gi|51011067 | Pyruvate kinase  (Pkm2b) | 0.50 | 67.38 | 7.6 | 21% | 140 | glycolysis |
| 11 | gi|60279651 | Betaine--homocysteine S-methyltransferase 1  (Bhmt) | 0.51 | 44.67 | 6.7 | 26% | 447 | methionine biosynthesis |
| 12 | gi|220678153 | Pyrophosphatase (Inorganic) 1  (Ppa1) | 0.48 | 33.22 | 5.2 | 23% | 108 | Oxidative phosphorylation |
| 13 | gi|37722017 | Ubiquitin C-terminal hydrolase L1  (Uchl1) | 0.42 | 24.63 | 5.2 | 34% | 201 | ubiquitin-dependent protein catabolism |
| 14 | gi|116325975 | ATP synthase subunit alpha, mitochondrial  (Atp5a1) | 0.23 | 59.88 | 9.1 | 10% | 369 | ATP biosynthesis, ion transport, ATP synthesis coupled proton transport |
| 15 | gi|41388972 | Phosphoglycerate kinase  (Pgk1) | 3.93 | 45.16 | 6.5 | 18% | 422 | phosphoglycerate kinase activity, glycolysis |
| 16 | gi|66773080 | ATP synthase subunit beta, mitochondrial  (Atpb) | 2.77 | 25.13 | 4.9 | 15% | 251 | ATP biosynthesis, ion transport, ATP synthesis coupled proton transport |
| 17 | gi|33585483 | Ubin protein  (Ubin) | 0.48 | 53.15 | 4.8 | 25% | 210 | protein modification |
| 18 | gi|41054373 | Calreticulin, like 2  (Calrl2) | 0.42 | 49.04 | 4.4 | 21% | 342 | calcium ion binding, protein folding |
| 19 | gi|46309515 | CLIP-associating protein 2  (Clasp2) | 0.31 | 141.2 | 9 | 8% | 59 | cell cycle, mitosis, cell division |
| 20 | gi|88930443 | Histone-binding protein RBBP4  (Rbb4) | 0.39 | 47.62 | 4.7 | 25% | 90 | DNA replication, chromatin remodeling, transcription, cell cycle, |
| 21 | gi|41054972 | 40S ribosomal protein SA  (Rpsa) | 0.48 | 23.68 | 6.9 | 27% | 292 | structural constituent of ribosome, protein biosynthesis |
| 22 | gi|41386743 | Eukaryotic translation elongation factor 2, like  (Eef2l) | 0.37 | 96.46 | 6.3 | 5% | 148 | translation elongation factor activity, GTPase activity, GTP binding |
| 23 | gi|41053764 | Thioredoxin-like 1  (Txnl1) | 0.49 | 32.44 | 4.8 | 33% | 383 | cell redox homeostasis |
| 24 | gi|47087315 | guanine nucleotide binding protein (G protein), beta polypeptide 1, like  (Gnb1l) | 0.43 | 38.08 | 5.6 | 13% | 164 | guanine nucleotide binding protein (G protein), beta polypeptide 1, like |
| 25 | gi|28502871 | Rho GDP dissociation inhibitor (GDI) alpha  (Arhgdia) | 0.42 | 23.15 | 5 | 41% | 374 | Rho GDP-dissociation inhibitor activity |
| 26 | gi|41055144 | Sorcin  (Sri) | 0.39 | 21.65 | 5.1 | 42% | 289 | calcium ion binding |
| 27 | gi|18859323 | 60S acidic ribosomal protein P0  (Rplp0) | 0.58 | 34.73 | 6.2 | 26% | 184 | embryonic development, translational elongation, ribosome biogenesis and assembly |
| 28 | gi|31419227 | Chaperonin containing TCP1, subunit 3  (Cct3) | 2.23 | 60.81 | 5.8 | 18% | 219 | protein folding, cellular protein metabolism |
| 29 | gi|32308151 | Annexin A1c  (Anxa1c) | 1.91 | 67.38 | 7.6 | 21% | 140 | calcium ion binding, calcium-dependent phospholipid binding |
| 30 | gi|18858947 | Type II basic cytokeratin  (Krt4) | 0.59 | 54299 | 5.4 | 22% | 387 | structural molecule activity |
| 31 | gi|6180207 | Type II cytokeratin  (Krt5) | 0.52 | 58551 | 5.3 | 34% | 475 | structural molecule activity |
| 32 | gi|18858519 | Type I cytokeratin, enveloping layer  (Cyt1) | 1.97 | 24286 | 4.8 | 64% | 405 | structural molecule activity |

Note. No. on gel, spot number as noted on the 2D gels; accession no., the Mascot results of the MALDI-TOF-MS/MS search of the NCBI nr database; fold change, the average fold changes as compared to the controls; Mw, molecular weight; pI, isoelectric point; match rate, the percentage of the number of mass values matched to the number of mass values searched; sequence coverage, the percentage sequence coverage of the hit; score, Mascot probability based on the Mowse score calculated for the MS/MS results; functional description: the biological processes in Gene Ontology terms. The average fold changes as compared to the controls. Signiﬁcance is reached at a score ≥ 40. A number > 1 indicates upregulation, and a number < 1 indicates downregulation.
